# Prediction of Partition Coefficients of Organic Compounds between SPME/PDMS and Aqueous Solution

**DOI:** 10.3390/ijms15022585

**Published:** 2014-02-14

**Authors:** Keh-Ping Chao, Yu-Ting Lu, Hsiu-Wen Yang

**Affiliations:** Department of Occupational Safety and Health, China Medical University, 91 Hsueh-Shih Rd., Taichung 40402, Taiwan; E-Mails: disney-14014@hotmail.com (Y.-T.L.); hwyang1224@gmail.com (H.-W.Y.)

**Keywords:** polydimethylsiloxane (PDMS), solid phase microextraction (SPME), partition coefficient, polarizability, molecular connectivity index

## Abstract

Polydimethylsiloxane (PDMS) is commonly used as the coated polymer in the solid phase microextraction (SPME) technique. In this study, the partition coefficients of organic compounds between SPME/PDMS and the aqueous solution were compiled from the literature sources. The correlation analysis for partition coefficients was conducted to interpret the effect of their physicochemical properties and descriptors on the partitioning process. The PDMS-water partition coefficients were significantly correlated to the polarizability of organic compounds (*r* = 0.977, *p* < 0.05). An empirical model, consisting of the polarizability, the molecular connectivity index, and an indicator variable, was developed to appropriately predict the partition coefficients of 61 organic compounds for the training set. The predictive ability of the empirical model was demonstrated by using it on a test set of 26 chemicals not included in the training set. The empirical model, applying the straightforward calculated molecular descriptors, for estimating the PDMS-water partition coefficient will contribute to the practical applications of the SPME technique.

## 1. Introduction

Solid phase microextraction (SPME) is a solvent-free sample preparation technique. The outer layer of the fused silica rod of the SPME device is coated with polymeric materials, such as polydimethylsiloxane. When the SPME device is placed in a sample matrix, the analyte is extracted and sorbed from the sample matrix onto the polymeric coating stationary phase. The analyte is then desorbed from the SPME device and the concentrated extract is analyzed using an instrument such as a gas chromatograph. The SPME technique simplifies the four steps of sampling, extraction, condensation and introduction of the sample into the analytical instrument [1]. In comparison to the traditional solid phase extraction methods, the advantages of SPME are lower cost, easy handling and shorter time procedures. Therefore, SPME has been widely used in the analysis of many organic compounds in water [2,3].

Sorption of the analyte between the sample matrix and the coating phase may be assumed to be an equilibrium partitioning process and may be modeled by a linear isotherm as follows:

(1) $$K_{fs}=\frac{C_{f}}{C_{s}}$$

where *C_f_* and *C_s_* are the equilibrium concentrations of the analyte in the coating and the sample matrix, respectively (M/L^3^); and *K_fs_* is the coating-matrix partition coefficient.

For an aqueous matrix, the total mass of analyte in the coating and the sample matrix can be represented as:

(2) $$C_{o}V_{s}=C_{f}V_{f}+C_{s}V_{s}=\left(K_{fs}V_{f}+V_{s}\right)C_{s}$$

where *C*_o_ is the initial concentrations of the analyte in the sample matrix (M/L^3^); and *V_f_* and *V_s_* are the volumes of the coating and the sample matrix, respectively (L^3^).

The mass of analyte sorbed by the coating of SPME, *M_f_* (M), can be determined as:

(3) $$M_{f}=C_{f}V_{f}=K_{fs}C_{s}V_{f}=\frac{K_{fs}C_{o}V_{s}V_{f}}{K_{fs}V_{f}+V_{s}}$$

Prior to using the SPME technique for sampling and analysis, the first step is to select the appropriate polymer as the SPME coated material to sorb the analyte. As shown in Equation 3, the mass of analyte sorbed by SPME is determined by the coating-matrix partition coefficient *K_fs_*. The proper application of SPME is primarily dependent on the partition coefficient of analyte between the polymeric coating and sample matrix. Therefore, there is a need to estimate the partition coefficient *K_fs_* for determining the sensitivity of SPME extraction [4–6].

Factors affecting the sorption of organic compounds onto the solid phase include electrical attraction, chemical affinity of the organic molecule, van der Waal’s force and the hydrophobic nature of the organic compound [7]. The physicochemical descriptors, such as polarizability, molecular connectivity index, and indicator variable, have been found to be successful in modeling the parameters for partition processes like octanol-water, octanol-air and air-water partition coefficients as well as adsorption coefficients and aqueous solubility of organic compounds [8–12].

This study is an attempt to develop predictive equations for the partition coefficients of a variety of chemicals in aqueous solutions onto SPME/PDMS based on their physicochemical properties and molecular structures. This was achieved by using the PDMS-water partition coefficients, *K_fw_*, retrieved from the literature [13,14] and relating them to their physicochemical descriptors, such as polarizability (Φ), molecular connectivity index (^1^χ), indicator variable (*I*), water solubility (*WS*), molecular weight (*M*_W_), and octanol-water partition coefficient (*log K*_ow_). The correlation analysis of *K_fw_* can provide an understanding in the partitioning process between SPME/PDMS and aqueous samples. Further, the predictive equations will provide a means of determination *K_fs_* for other chemicals, and facilitate the application of the SPME technique.

## 2. Results and Discussion

### 2.1 Correlation Analysis

PDMS is a hydrophobic and nonpolar polymer. Therefore, the nonpolar attractive force causing physical adsorption of organic compounds onto PDMS surfaces is the van der Waals force. One of the attractive potential energies for the van der Waals forces is the dipole-induced dipole interaction, also called London dispersion force, between PDMS and organic molecules. When SPME/PDMS is placed in the aqueous solution, the dipole moment of PDMS creates an electric field which polarizes the charges on organic molecules. The magnitude of induced change in dipole moment is determined by the polarizability of organic molecules [15]. As presented in Table 1, *K_fw_* was found to correlate best with the polarizability of organic compounds (*r* = 0.977, *p* < 0.05). Therefore, the polarizability can be a good basis to understand the partition between SPME/PDMS and organic compounds in the aqueous solution.

**Figure 1.**
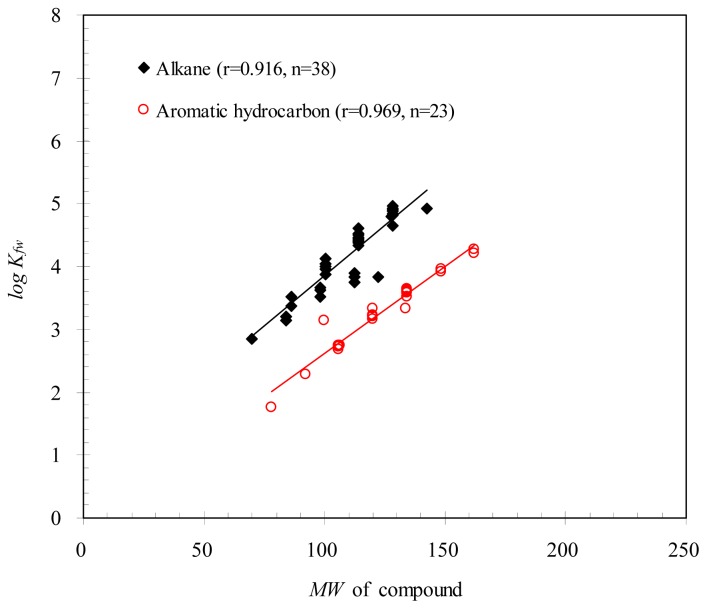
Correlations of *K_fw_* for the training set with their molecular weights.

In general, the van der Waals forces of molecules increase with an increase in their molecular weights [7]. Table 1 shows that *K_fw_* was proportional to the molecular weights of organic compounds but only with a correlation coefficient *r* = 0.474 (*p* < 0.05). If the organic compounds were grouped in different categories, *i.e.*, alkanes and aromatic hydrocarbons, for correlation analysis, Figure 1 indicates that *K_fw_* was significantly correlated to their molecular weights (*r* ≥ 0.916). This result may imply that diffusion of organic molecules in the PDMS fiber was not a governing mechanism for the mass transfer between PDMS and organic compounds.

According to the principle of “like dissolves like”, it is plausible that a non-polar compound is more easily sorbed by PDMS. In general, the larger the octanol-water partition coefficient of a chemical, the lower the polarity. Several researchers indicated that *log K_fw_* of SPME/PDMS linearly increased with *log K*_ow_ for organic compounds [16,17]. As seen in Table 1, *log K_fw_* for the training set showed a positive correlation with their *log K*_ow_ (*r* = 0.774, *p* < 0.05). On the other hand, an inversely proportional trend was observed between *log K_fw_* and water solubility (*r* = 0.559, *p* < 0.05), indicating that the less soluble compound is more likely partitioned to SPME/PDMS than more soluble compounds.

The molecular connectivity index conveys the degree of branching for the molecular structure [18]. The greater the degree of branching in a molecule, the lower will be the value of the molecular connectivity index. Table 1 shows that *K_fw_* was well proportional to their molecular connectivity indexes with a correlation coefficient *r* = 0.743 (*p* < 0.05). Several researchers have indicated that the molecular connectivity indexes were significantly correlated to polarizability [12,18]. In this study, the molecular connectivity indexes of the training set were proportional to their polarizability (*r* = 0.676, *p* < 0.05). However, it is speculated that the branch molecule is less easily partitioned to SPME/PDMS.

### 2.2 Empirical Models

The best predictive model for *K_fw_* was obtained through the multiple regression analysis using a statistical software package SPSS 20.0 version (IBM, New York, NY, USA, 2011). Based on the stepwise regression with 95% confidence limits, the multiple regression analyses of *K_fw_* was as follows (*r* = 0.99, *p* < 0.05):

(4) log Kfw=1.545+0.724Φ+0.292 χ1-0.227 I+0.001WS

The above model explained 98% of the variance in the *K_fw_* data of the training set. Nirmalakhandan and Speece [12] developed a quantitative structure-activity relationship (QSAR) model for estimating Henry’s constant. In their study, the best predictive equation consisted of the molecular connectivity index, polarizability, and indicator variable of organic compounds. For the physicochemical descriptors of Equation (4), Table 1 indicates that the water solubility (*r* = 0.559) and the indicator variable (*r* = 0.583) were correlated to *K_fw_* with the lower correlation coefficients. If the water solubility and the indicator variable were excluded for Equation (4), the multiple regressions of *K_fw_* were as follows:

(5) log Kfw=1.447+0.728Φ+0.316 χ1-0.224 I

(6) log Kfw=1.502+0.918Φ+0.177 χ1

**Figure 2.**
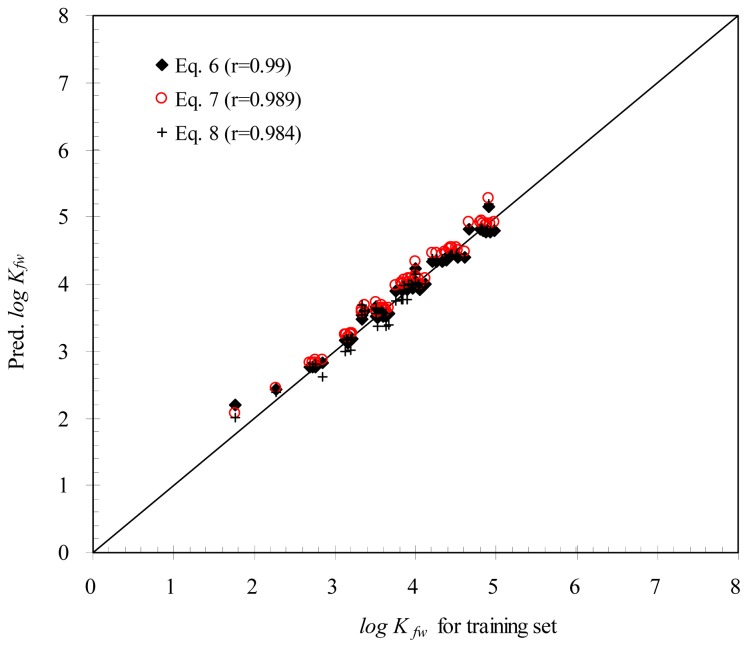
Comparison of *K_fw_* between the training set and predictive models.

For Equations (5) and (6), the correlation coefficients (*r*) were 0.989 and 0.984, respectively, which were similar to that of Equation (4). Figure 2 presents a comparison of *K_fw_* for the training set with *K_fw_* predicted from Equations (4)–(6). Figure 2 shows that the agreement between the training set and the models predicted *K_fw_* is very satisfactory.

Several researchers have used the linear solvation energy relationship (LSER) method to estimate *K_fw_* for SPME [19,20]. The LSER model consists of five descriptors of chemicals, *i.e.*, molar refraction, polarizability, hydrogen bond acidity, hydrogen bond basicity, and McGowan characteristic volume (mainly representing London dispersion). As compared with the models developed herein, the physicochemical descriptors for the LSER method require a large data set. In particular, the physicochemical property data for the LSER method may be unavailable for the compound of interest [16,21].

**Figure 3.**
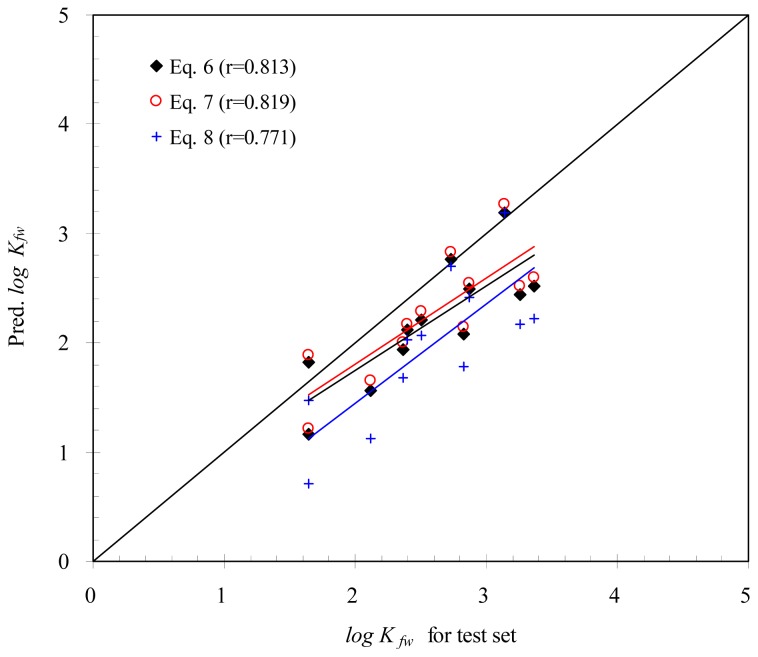
Comparison of *K_fw_* between the test set and predictive models.

To assess the validity of the above empirical models, *K_fw_* for the organic compounds of the test set was predicted using Equations (4)–(6). Figure 3 indicates a comparison of *K_fw_* for the test set with *K_fw_* predicted using the empirical models. As shown in Figure 3, most of *K_fw_* for the test set were greater than those predicted using the empirical models. The slopes of regression analysis shown in Figure 3 implied that there was a systematic difference in the experimental measurement of *K_fw_* between the training and test sets. For example, Table 3 indicates that the values of *K_fw_* were 566 and 564 for ethylbenzene and *p*-xylene, respectively. In the study of Xia *et al.* [14], *K_fw_* was 512 and 575 for ethylbenzene and *p*-xylene which were not included in the test set. However, the correlation coefficients of *K_fw_* were 0.813, 0.819 and 0.771 for Equations (4)–(6), respectively. For a practical purpose, the regression equation, consisting of the molecular connectivity index, polarizability, and indicator variable, can be an adequate model to predict *K_fw_* for SPME/PDMS.

## 3. Method

### 3.1. PDMS-Water Partition Coefficients

The training set of PDMS-water partition coefficients for alkanes and aromatic hydrocarbons was compiled from the literature [13] published by Dr. Pawliszyn who invented the SPME technique. As shown in Table 2, the values of *K_fw_* (*n* = 61) exhibit a wide range from 58 (for benzene) to 82,430 (for 3,3-dimethyloctane). In addition, the organic compounds of the training set have a wide range of water solubility, e.g., 1.4 × 10^−2^ mg/L for 1,3,5-triethylbenzene, and 1.79 × 10^3^ mg/L for benzene. The test set of PDMS-water partition coefficients (*n* = 26), not included in the training set, was retrieved from the study of Xia *et al.* [14]. Table 3 indicates that the magnitude of *K_fw_* values for the test set, consisting of alcohols, benzenes and phenols, were between 10^−1^ and 10^3^. *K_fw_* for the training and test sets were experimentally obtained using the SPME/PDMS devices at 25 °C.

### 3.2. Physicochemical Descriptors

In this study, the polarizability and molecular connectivity index were used to represent the interactions between the analyte and PDMS coating. As shown in Equation (7), the polarizability *Φ* was determined by the addition of atom/bond contribution factors [12,22].

(7) $$\Phi =\sum F_{i}\times (number\;of\;atom/bond)$$

where *F_i_* is the contribution factors indicated in Table 4.

The molecular connectivity index is a topological descriptor of molecular structure based on a count of skeletal atom groupings of a chemical compound [18,23]. For each atom of a molecule, a δ value is assigned as the difference between the number of valence electrons and the number of hydrogen atoms attached to that atom. In this study, the first-order connectivity index, ^1^χ, was calculated as follows [9,11,12]:

(8) χ1=∑q=1n(δiδj)q-0.5

where δ*_i_* and δ*_j_* are the δ values of two adjacent atoms *i* and *j*, respectively; and *n* is the number of bonds in the molecule.

An indicator variable, *I*, was used to differentiate between compounds on the basis of their ability to take place in hydrogen bonding [24]. *I* was assigned a value of 1 for all compounds containing an electronegative element, such as oxygen, nitrogen, and halogen *etc.*, attached directly to a carbon atom holding a hydrogen atom. In addition, the value of *I* was assigned 1 for acetylinic compounds and aromatic compounds with partially substituted hydrogen atoms. For the other compounds, *I* was set equal to zero [12].

## 4. Conclusions

From the single-parameter model analyses of the training set, the PDMS-water partition coefficients were proportional to the molecular connectivity index, polarizability, molecular weight, and octanol-water partition coefficient of organic compounds, while an inversely proportional trend was observed between *K_fw_* and the water solubility as well as the indicator variable. *K_fw_* was significantly dependent on the polarizability of the organic compounds. Based on the results of multiple regression analyses, several correlations for *K_fw_* were developed using the polarizability, molecular connectivity index, indicator variable and water solubility, indicating a good agreement (*r* ≥ 0.771) between the training set and the predicted *K_fw_*. In addition, the correlations were able to adequately predict *K_fw_* for a test set which were not included in the correlation development. The empirical model developed in this work is more versatile than current available correlations. In order to broaden the predictive abilities, however, future work should be conducted to calculate *K_fw_* using the empirical model for a variety of organic compounds.

## Figures and Tables

**Table 1.** Correlation analysis of *K_fw_* for the training set.

Descriptor	Correlation
Φ	*log K_fw_* = 1.933 + 1.026 *Φ* (*r* = 0.977, *p* < 0.05)
*log K*_ow_	*log K_fw_* = 0.319 + 0.881 *log K*_ow_ (*r* = 0.774, *p* < 0.05)
^1^χ	*log K_fw_* = 0.750 + 0.862 *^1^*χ (*r* = 0.743, *p* < 0.05)
*I*	*log K_fw_* = 4.144 − 0.846 *I* (*r* = 0.583, *p* < 0.05)
*WS*	*log K_fw_* = 3.936 − 0.002 *WS* (*r* = 0.559, *p* < 0.05)
*M*_W_	*log K_fw_* = 1.859 + 0.017 *M*_W_ (*r* = 0.474, *p* < 0.05)

**Table 2.** The training set of PDMS-water partition coefficients and physico-chemical descriptors.

Compound	*K_fw_*	^1^χ	Φ	*I*	*M*_W_ †	*WS* ‡	*log K*_ow_
Benzene	58	2	0.165	1	78	1790	2.13
Toluene	189	2.411	0.502	1	92	526	2.73
*o*-Xylene	485	2.827	0.839	1	106	178	3.12
*m*-Xylene	533	2.821	0.839	1	106	161	3.2
*p*-Xylene	564	2.821	0.839	1	106	162	3.15
Ethylbenzene	566	2.971	0.839	1	106	169	3.15
Cyclopentane	712	2.5	0.733	0	70	156	3.01
Methylcyclopentane	1356	2.894	1.07	0	84	42	3.37
Isopropylbenzene	1412	3.354	1.176	1	120	61.3	3.66
1,3,5-Trimethylbenzene	1451	3.232	1.176	1	120	48.2	3.42
1-Methyl-4-Ethylbenzene	1581	3.382	1.176	1	120	94.9	3.63
Cyclohexane	1592	3	1.07	0	84	55	3.44
*n*-Propylbenzene	1664	3.471	1.176	1	120	52.2	3.69
1,2,4-Trimethylbenzene	2183	3.238	1.76	1	120	57	3.63
*tert*-Butylbenzene	2185	3.661	1.513	1	134	29.5	4.11
2,3-Dimethylbutane	2359	2.643	1.782	0	86	22.5	3.42
2-Methylpentane	3224	2.77	1.782	0	86	14	3.21
3-Methylpentane	3270	2.808	1.782	0	86	17.9	3.6
1-Methyl-3-Isopropylbenzene	3284	3.765	1.513	1	134	42.5	4.5
*trans*-1,2-Dimethylcyclopentane	3372	3.207	1.407	0	98	33.9	3.52
1-Methyl-3-*n*-Propylbenzene	3772	3.882	1.513	1	134	9.09	4.67
*n*-Butylbenzene	3872	3.971	1.513	1	134	11.8	4.38
1,2-Dimethyl-4-Ethylbenzene	3984	3.8	1.513	1	134	12.7	4.5
*sec*-Butylbenzene	4011	3.892	1.513	1	134	17.6	4.57
Isobutylbenzene	4197	3.827	1.513	1	134	10.1	4.68
*cis*-1,3-Dimethylcyclopentane	4289	3.288	1.407	0	98	33.9	3.52
1,3-Dimethyl-2-Ethylbenzene	4345	3.805	1.513	1	134	19.6	4.28
Methylcyclohexane	4657	3.394	1.407	0	98	14	3.61
*cis*-*trans*-*cis*-1,2,4-Trimethylcyclopentane	5621	3.698	1.744	0	112	14.8	3.94
*trans*-1,2-Dimethylcyclohexane	6638	3.805	1.744	0	112	5.2	4.01
1-Ethyl-1-methylcyclopentane	6831	3.768	1.744	0	122	11.6	4.05
2,2-Dimethylpentane	7349	3.061	2.119	0	100	4.4	3.67
*cis*-1,2-Dimethylcyclohexane	7826	3.805	1.744	0	112	6	4.01
*n*-Pentylbenzene	8195	4.471	1.85	1	148	3.37	4.9
2,4-Dimethylpentane	8989	3.126	2.119	0	100	5.5	3.63
2-Methylbutylbenzene	9099	4.365	1.85	1	148	12.7	4.43
2,2,3-Trimethylbutane	9802	3.944	2.119	0	100	28.9	3.59
2-Methylhexane	10,202	3.27	2.119	0	100	2.54	3.71
3,3-Dimethylpentane	10,963	3.121	2.119	0	100	5.92	3.67
3-Methylhexane	11,146	2.9	2.119	0	100	4.95	3.71
2,3-Dimethylpentane	13,074	3.181	2.119	0	100	5.25	3.63
1,2,4-Triethylbenzene	16,253	4.92	2.187	1	162	2.9	5.11
1,3,5-Triethylbenzene	18,517	4.914	2.187	1	162	0.014	5.11
2,2,3-Trimethylpentane	21,205	3.481	2.456	0	114	2.4	4.09
2,5-Dimethylhexane	23,519	3.626	2.456	0	114	9.2	4.12
2,2-Dimethylhexane	24,504	3.561	2.456	0	114	0.2	4.16
2-Methylheptane	25,806	3.77	2.456	0	114	7.97	4.2
4-Methylheptane	27,274	3.808	2.456	0	114	7.97	4.2
3-Ethylhexane	28,370	3.846	2.456	0	114	7.97	4.2
3-Methylheptane	31,856	3.808	2.456	0	114	0.792	4.2
2,3-Dimethylhexane	33,749	3.681	2.456	0	114	9.2	4.12
2,4-Dimethylhexane	41,133	3.664	2.456	0	114	9.87	4.12
2-Methyloctane	45,267	4.27	2.793	0	128	2.87	4.69
3,3-Diethylpentane	63,718	4.243	2.793	0	128	2.9	4.65
3-Methyloctane	66,682	4.308	2.793	0	128	2.87	4.69
2,3-Dimethylheptane	68,675	4.181	2.793	0	128	3.37	4.61
3,3-Dimethylheptane	76,013	4.121	2.793	0	128	2.9	4.65
3,5-Dimethylheptane	78,829	4.202	2.793	0	128	3.11	4.61
3,3-Dimethyloctane	82,430	4.621	3.13	0	142	2.9	5.14
2,5-Dimethylheptane	84,142	4.164	2.793	0	128	3.11	4.61
3,4-Dimethylheptane	93,292	4.219	2.793	0	128	3.11	4.61

† *M*_W_: g/mole;

‡ *WS*: mg/L.

**Table 3.** The test set of PDMS-water partition coefficients.

Compound	*K_fw_*	^1^χ	Φ	*I*	*WS* ‡
Benzyl alcohol	0.447	2.580	−0.323	1	4.29 × 10^4^
4-Fluorophenol	0.525	2.234	−1.110	1	1.25 × 10^4^
Phenol	0.661	2.134	−0.540	1	8.28 × 10^4^
*m*-Cresol	0.933	2.545	−0.323	1	2.27 × 10^4^
Phenethyl alcohol	1.318	3.081	0.014	1	2 × 10^4^
3-Methylbenzyl alcohol	1.479	2.991	0.014	1	5 × 10^4^
3-Chlorophenol	2.042	2.647	−0.727	1	2.6 × 10^4^
3,5-Dimethylphenol	2.630	2.956	0.014	1	4.88 × 10^3^
3-Bromophenol	2.884	3.026	−0.762	1	2.4 × 10^4^
4-Ethylphenol	3.981	3.106	0.014	1	4.9 × 10^3^
4-Chloroaniline	6.918	2.676	−0.022	1	3.9 × 10^3^
Phenyl acetate	7.244	3.023	−1.430	1	4.64 × 10^3^
Benzonitrile	10.96	2.384	0.753	1	2 × 10^3^
Acetophenone	10.96	2.865	−0.605	1	6.13 × 10^3^
4-Chloroacetophenone	43.7	3.342	−0.672	1	111
Methyl benzoate	44.7	2.977	−1.430	1	2.1 × 10^3^
Ethylbenzoate	131.8	3.565	−1.093	1	720
4-Chloroanisole	234.4	3.036	−0.390	1	237
Chlorobenzene	251.2	2.477	0.098	1	498
Bromobenzene	323.6	2.891	0.063	1	410
Iodobenzene	537.1	3.161	0.692	1	340
Naphthalene	676.1	3.405	−0.344	1	31
4-Chlorotoluene	741.3	3.095	0.435	1	106
Propylbenzene	1380	3.471	1.176	1	52.2
1-Methylnaphthalene	1819	3.821	−0.007	1	25.8
Biphenyl	2344	4.071	−0.007	1	6.9

‡ *WS*: mg/L.

**Table 4.** Contribution factors for the calculation of polarizability.

Atom/bond	Contribution factor	Atom/bond	Contribution factor
Carbon	0.577	Iodine	0.407
Hydrogen *	−0.120	Fluorine	−0.570
Oxygen	−0.825	Cycle	−0.952
Hydroxyl	−3.701	Double bond	−0.859
Chlorine	−0.187	Triple bond	−0.109
Bromine	−0.222	-	-

Data was obtained from Nirmalakhandan and Speece [12];

* Attached to carbon atoms only.
